# Introducing npj Imaging: a new journal to serve the bio- and medical imaging communities

**DOI:** 10.1038/s44303-024-00015-5

**Published:** 2024-04-08

**Authors:** Timothy H. Witney

**Affiliations:** School of Biomedical Engineering & Imaging Sciences, King’s College London, London, UK

The ability to see inside the living human body and image the function of cells within it has revolutionised our understanding of disease. From magnetic resonance imaging (MRI) of soft tissues to complex super-resolution images of actin filaments in metastatic tumour cells, imaging provides a cornerstone of modern medicine, fuelling basic and translational discoveries. It’s the combination of exquisite structural and functional information obtained across cellular and whole-body scales that makes imaging such a unique discipline.

Historically, the fields of bioimaging and medical imaging have developed separately. The advanced facilities required for both fields are typically not co-located; consequently, the cross-fertilisation of ideas and technologies often does not occur as frequently as we would like. To address this need, we are excited to launch *npj Imaging*, a new open-access journal dedicated to publishing impactful and high-quality content on all aspects of imaging research across scales and the translational pipeline. The expertise of our Associate Editors, who handle the manuscripts we receive, span these disciplines, supported by our excellent Editorial Board.

Our journal provides a platform for imaging researchers to showcase their latest cutting-edge discoveries to the community. As imaging is inherently multidisciplinary, our journal accepts submissions from teams working at the interface of medical, biological, and physical sciences, and the application of artificial intelligence to imaging research. The breadth of the journal’s scope is exemplified in the first ten papers that we have published since our first paper landed in late November 2023. This paper came from the lab of Reto Fiolka and described a novel method to image the entire cell at high spatial resolution using a technique called oblique plane structured illumination microscopy (OPSIM)^1^. At *npj Imaging*, we are interested in receiving manuscripts which describe new probes or techniques, but these discoveries must have a biological application. By using OPSIM, cells could be imaged at a speed which permitted the assessment of mitochondria and ER dynamics. Other examples of innovative imaging used to improve our understanding of (patho)physiology includes the use of new Raman topography imaging workflow for the surgical resection of tumours^2^ and the development of self-assembled peptide-dye nanostructures for in vivo tumour imaging^3^.

Bringing together both bioimaging and medical imaging is not without its challenges. Each imaging technique comes with its unique set of limitations, such as spatial or temporal resolution, tissue and data processing requirements, or the specificity of the imaging signal observed. We encounter further challenges when we seek to combine different imaging techniques, as highlighted in our recent perspective ‘Multimodal bioimaging across disciplines and scales: challenges, opportunities and breaking down barriers’^4^. Despite these barriers, the combination of multiple imaging techniques has the potential to overcome the inherent restrictions of individual modalities to gain a deeper understanding of disease.

At *npj Imaging* we are always looking for ways to improve data visualisation; we are an imaging journal after all! Figures or figure parts are not restricted to static images but can be submitted as movies. Examples could include a fly-through of super-resolution microscopy images, or as we have published recently^5^, a rotating three-dimensional positron emission tomography maximum intensity projection. Furthermore, being an open-access journal means that all published papers are made immediately available for all to read. I look forward to working closely with the fantastic imaging community over the coming years as a champion of the field and of your work. It is an honour to serve as *npj Imaging*’s first Editor-in-Chief.
